# Impact of seed amplification assay and surface-enhanced Raman spectroscopy combined approach on the clinical diagnosis of Alzheimer’s disease

**DOI:** 10.1186/s40035-023-00367-9

**Published:** 2023-07-12

**Authors:** Cristiano D’Andrea, Federico Angelo Cazzaniga, Edoardo Bistaffa, Andrea Barucci, Marella de Angelis, Martina Banchelli, Edoardo Farnesi, Panagis Polykretis, Chiara Marzi, Antonio Indaco, Pietro Tiraboschi, Giorgio Giaccone, Paolo Matteini, Fabio Moda

**Affiliations:** 1 Institute of Applied Physics “Nello Carrara”, National Research Council, 50019 Sesto Fiorentino, Italy; 2 Division of Neurology 5 and Neuropathology, Fondazione IRCCS Istituto Neurologico Carlo Besta, 20133 Milan, Italy; 3 Institute of Physical Chemistry (IPC) and Abbe Center of Photonics (ACP), Friedrich Schiller University Jena, 07743 Jena, Germany; 4 Leibniz Institute of Photonic Technology, 07745 Jena, Germany

**Keywords:** Alzheimer’s disease, Amyloid-β protein, Seed amplification assay, Surface-enhanced Raman spectroscopy

## Abstract

**Background:**

The current diagnosis of Alzheimer’s disease (AD) is based on a series of analyses which involve clinical, instrumental and laboratory findings. However, signs, symptoms and biomarker alterations observed in AD might overlap with other dementias, resulting in misdiagnosis.

**Methods:**

Here we describe a new diagnostic approach for AD which takes advantage of the boosted sensitivity in biomolecular detection, as allowed by seed amplification assay (SAA), combined with the unique specificity in biomolecular recognition, as provided by surface-enhanced Raman spectroscopy (SERS).

**Results:**

The SAA-SERS approach supported by machine learning data analysis allowed efficient identification of pathological Aβ oligomers in the cerebrospinal fluid of patients with a clinical diagnosis of AD or mild cognitive impairment due to AD.

**Conclusions:**

Such analytical approach can be used to recognize disease features, thus allowing early stratification and selection of patients, which is fundamental in clinical treatments and pharmacological trials.

**Supplementary Information:**

The online version contains supplementary material available at 10.1186/s40035-023-00367-9.

## Introduction

Alzheimer's disease (AD) is the most common neurodegenerative disorder in the elderly with an incidence that is progressively increasing worldwide [1]. The main neuropathological hallmark of AD is the presence of two protein aggregates, extracellular amyloid plaques made up of amyloid-β protein (Aβ) and intracellular neurofibrillary tangles made up of hyperphosphorylated tubulin-associated unit (tau) protein [2, 3]. Before aggregating, both proteins undergo conformational rearrangements which increase their propensity to form the characteristic insoluble assemblies. Although several target proteins and risk factors contribute to AD etiology, Aβ seems to play a significant role and is considered the earliest and main pathological actor [4–6]. Aβ is derived from the proteolysis of the amyloid precursor protein [7]. Upon misfolding, Aβ acquires pathological properties, spreads throughout the brain and triggers a cascade of neurotoxic events, ultimately leading to neurodegeneration [8–11]. Remarkably, the size, the morphology and the localization of Aβ aggregates differ considerably in the brains of AD patients: this strengths the evidence that the disease is phenotypically heterogeneous, and such heterogeneity likely correlates with structural diversities of Aβ species [12–17]. Therefore, characterization of Aβ aggregates in the brain enables classification of AD in different subgroups [17].

At present, the clinical diagnosis of AD mostly relies on the NIA-AA (National Institute of Aging – Alzheimer’s Association) criteria that were proposed in 2011 and subsequently revised. However, the definite diagnosis still requires a series of neuropathological examinations [18, 19]. This is partially due to the fact that the clinical, laboratory and instrumental biomarkers are not strictly specific for AD and can be altered in other neurodegenerative conditions [20]. Therefore, there is a need for more specific, cost-effective, easy-to-identify and reliable biomarkers to improve the clinical diagnostic accuracy of AD, eventually enabling the early identification of disease phenotypes.

By means of a seed amplification assay (SAA) technique, the presence of pathological Aβ species (typically found in the brain) in the cerebrospinal fluid (CSF) of AD patients has been demonstrated [21]. In particular, SAA amplifies small amount of Aβ oligomers in biological fluids at the expense of synthetic Aβ peptides which are used as the reaction substrate. The reaction leads to the formation of Aβ amyloid fibrils which is monitored by thioflavin-T (ThT) fluorescent dye. From another point of view, the most recent advancements in the optical field led to the possibility of developing effective spectroscopy systems for a label-free description of Aβ species [22–26]. In particular, Raman spectroscopy is a label-free, non-invasive and non-destructive vibrational technique that provides the molecular fingerprint of biomolecules [27, 28]. Taking advantages of the addition of plasmonic nanostructures, surface-enhanced Raman spectroscopy (SERS) overcomes the Raman spectroscopy detection limits, pushing the biorecognition sensitivity down several order of magnitudes [29, 30]. As an example, SERS proved powerful in distinguishing small  concentrations of specific aggregated forms of neurodegenerative biomarkers and in postulating a correlation between their molecular structure and neurotoxicity [31–34].

In this work, we set up a modified SAA protocol and combined it with SERS (SAA-SERS) for the innovative analysis of CSF collected from well-characterized patients with a clinical diagnosis of AD, mild cognitive impairment due to AD (MCI-AD) or other neurological conditions (ONC). The aim was to evaluate whether the proposed combined approach could prove effective in improving AD clinical diagnosis, eventually allowing patient stratification. These analyses were supported by machine learning and finally correlated with the available clinical, instrumental and laboratory findings.

## Materials and methods

### Collection of CSF samples

CSF samples collected from enrolled patients were centrifuged at 1000 × *g* for 10 min and stored in polypropylene tubes (Sarstedt, Nümbrecht, Germany) at − 80 °C until analysis. The demographic and neuropsychological data as well as the available laboratory findings of the patients included in the study are summarized in Table 1.

**Table 1:** Demographic, laboratory and neuropsychological data

Patient code	Clinical diagnosis	Age at CSF collection (years)	p-tau (pg/ml) *	t-tau (pg/ml) **	Aβ_1-42_ (pg/ml) ***	Aβ_1-40_ (pg/ml) ****	Aβ_1-42_/Aβ_1-40_ *****	MMSE score
AD#1	AD	63	56	452	694	n.a	n.a	22/30
AD#2	AD	58	98	1195	464	n.a	n.a	21/30
AD#3	AD	65	114	761	477	n.a	n.a	21/30
AD#4	AD	54	138	1133	349	9376	0.037	n.a
AD#5	AD	57	74	513	267	4661	0.057	12/30
AD#6	AD	49	64	427	525	n.a	n.a	26/30
AD#7	MCI-AD	52	72	451	575	n.a	n.a	28/30
AD#8	AD	75	79	779	464	n.a	n.a	23/30
AD#9	AD	63	52	404	483	n.a	n.a	n.a
AD#10	AD	75	179	1434	436	n.a	n.a	26/30
AD#11	AD	62	58	430	246	4603	0.053	21/30
AD#12	AD	77	94	855	314	6581	0.048	20/30
AD#13	MCI-AD	75	115	1130	440	8292	0.053	28/30
AD#14	MCI-AD	79	93	905	394	7536	0.052	28/30
AD#15	AD	70	60.1	396	230	4095	0.056	18/30
AD#16	AD	69	214.6	1258	261	7875	0.033	24/30
AD#17	AD	70	181.5	1211	577	10,190	0.057	24/30
AD#18	AD	74	239	1387	218	3839	0.057	26/30
AD#19	AD	74	93.2	610	434	9355	0.046	22/30
AD#20	MCI-AD	84	93.1	469	361	9005	0.040	29/30
ONC#1	PSP	71	46	278	460	7750	0.046	n.a
ONC#2	PD	58	24.1	131	592	6333	0.094	n.a
ONC#3	HyC	77	29	229	801	9760	0.082	n.a
ONC#4	HyC	71	28.7	163	594	7499	0.079	n.a
ONC#5	HyC	75	12.7	95	147	2090	0.070	n.a
ONC#6	HyC	78	54.4	299	645	10,337	0.062	n.a
ONC#7	HyC	75	21.7	136	267	4150	0.064	n.a
ONC#8	HyC	44	19.1	77	382	4374	0.087	n.a
ONC#9	HyC	63	n.a	n.a	163	1771	0.090	n.a
ONC#10	HyC	74	20.1	144	461	5149	0.090	n.a
ONC#11	HyC	59	26	138	1296	8101	0.160	n.a

*Measured with the INNOTEST® PHOSPHO-TAU (181P). Normal reference value < 61 pg/ml [50]

**Measured with the INNOTEST® hTAU Ag. Normal reference values: < 300 pg/ml, 21–50 years; < 400 pg/ml, 51–70 years; < 500 pg/ml, 71–93 years [51]

***Measured with the Lumipulse® G β-Amyloid 1–42. Normal reference value > 640 pg/ml [52]

****Measured with the Lumipulse® G β-Amyloid 1–40. Normal reference value range 7755–16,715 pg/ml [52]

***** Normal reference value range 0.068–0.115. Lower levels may be indicative or predictive of AD pathology [53]

n.a.: not available

### Standard laboratory analyses of CSF samples

CSF samples were analyzed with LUMIPULSE® G600II instrument (Fujirebio, Ghent, Belgium) to evaluate the concentrations of amyloid-β 1–42 (Aβ_1-42_) and amyloid-β 1–40 (Aβ_1-40_), as previously described [35, 36] or with ELISA (Fujirebio) to determine the concentrations of total tau (t-tau; INNOTEST® hTAU Ag) and phosphorylated tau 181 (p-tau; INNOTEST® PHOSPHO-TAU (181P)).

### SAA analyses of CSF samples

Each CSF sample was subjected to SAA analyses using two reaction mixes: (1) Mix 1: Tris–HCl 100 mM, synthetic Aβ_1-40_ peptide 10 µM (Life Technologies, Carlsbad, CA) and ThT 10 µM for evaluating the SAA aggregation kinetics; (2) Mix 2: Tris–HCl 100 mM and synthetic Aβ_1-40_ peptide 10 µM (Life Technologies) for SERS and atomic force microcopy (AFM) analyses. A single batch of Aβ_1-40_ peptide (purity > 95%, ThermoFisher Scientific, Waltham, MA) was dissolved in NaOH 10 mM (Merck, Darmstadt, Germany) to a final concentration of 230 μM and used for the analyses (Additional file 1: Fig. S1a, b). Ninety microliters of each reaction mix (Mix 1 or Mix 2) was placed in a black 96-well optical flat-bottom plate (Thermo Scientific) and supplemented with 10 µl of CSF samples reaching a final volume of 100 µl. Each CSF sample was analyzed in triplicate for each experimental condition and for at least two times by different operators. The plates were sealed with sealing films (ThermoFisher Scientific), inserted into a FLUOstar OMEGA microplate reader (BMG Labtech, Ortenberg, Germany) and subjected to shaking cycles of 1 min (600 rpm, double orbital) followed by 14 min incubation at 35 °C. For evaluating the aggregation kinetics, the average fluorescence intensity of the three replicates of each sample (analyzed using Mix 1) was calculated and plotted against time together with the standard error of the mean (mean ± SEM). A threshold of 75 h and 550 fluorescence arbitrary unit (AU) was set to discriminate seed-competent and seed-incompetent samples. For SERS and AFM experiments, the three replicates of each sample were pooled together and stored at − 80 °C before analysis.

### AFM

The morphology of the SAA products was examined using tapping-mode AFM. An aliquot of sample solution (3 μl) was dried on top of freshly cleaved mica at 32 °C for 90 min, followed by 2 rinsing cycles in Milli-Q water (100 μl) to remove salts and debris and drying under a gentle nitrogen steam. Samples were then imaged using a JPK NanoWizard III Sense (Bruker, Germany) scanning probe microscope operated in AFM mode. Single-beam uncoated silicon cantilevers (HQ:NSC15/Cr-Au BS, MikroMasch, Germany) were used, with a force constant of 40 N/m. Drive frequency was between 250 and 300 kHz, and the scan rate was 0.4 Hz.

### SERS analysis of SAA products

All SAA reaction products were collected at 150 h and analyzed using a SERS-active substrate based on networks of silver nanowires (AgNWs), as recently reported [37, 38]. Briefly, 2 ml of AgNWs/isopropyl alcohol was micro-filtered under nitrogen pressure through a polytetrafluoroethylene (PTFE) membrane (Sartorius, pore 0.45 µm) by using an Amicon Stirred Cell (Millipore, Model 8003, total volume 3 ml) and blocked on its top forming a 10-µm-thick layer of intertwined wires. The AgNWs@PTFE substrate was then patterned in 10 isolated spots of 1 mm diameter by a laser engraver (NEJE, λ = 405 nm, max power 3 W, spatial resolution 0.45 µm) (Fig. S1c). Immediately after it was thawed out to room temperature (RT), 2 µl of SAA product solution was drop-casted on a SERS-active spot, dried in air, rinsed twice with 2 µl of ultrapure water for 1 min in order to remove any residual trace of Tris buffer, and finally dried at RT.

SERS spectra were acquired using an XPlora micro-Raman spectrometer (Horiba, Montpellier, France) working in backscattering geometry, with an excitation wavelength at 785 nm, focused through a 10 × objective (Olympus, 0.25 NA, 7 µm waist) and laser power at the sample of 0.6 mW. For each sample a total of 50 spectra on different positions within an area of 0.24 mm^2^ (600 × 400 µm^2^) of the AgNWs@PTFE spot were collected. Each spectrum was acquired in the range of 950–1740 cm^−1^, illuminating the sample for 10 s of integration time, dispersing the scattered light by a 1200 grooves/mm grating, and collecting it with a Peltier cooled CCD detector (Horiba, France). All the spectra were corrected in wavelength, by acquiring the spectrum of a bulk crystalline silicon sample and calibrating the grating at the beginning of each measurement session with the first-order Raman peak of c-Si (520.8 cm^−1^). Each SERS experiment was conducted in two replicates by different operators.

In order to exclude any signal fluctuations due to operational factors (local inhomogeneities of the AgNWs@PTFE substrate, changes in laser focusing or in autofluorescence background) and to appreciate small signal variations in ONC and AD samples, a pre-processing of the data was performed before their evaluation by means of a well-established analytical pipeline reported in literature [38, 39]*.* The spectra were corrected for cosmic ray spikes, baselined (polynomial fit), smoothed and area normalized by using the Labspec 6 software (Horiba) (Additional file 1: Fig. S2).

### Machine learning analysis

Initially, SERS spectra were analyzed using t-distributed stochastic neighbor embedding (t-SNE) algorithm to obtain a bi-dimensional view of spectra distribution across all patients in the study [40]. Afterward, a pattern classifier was trained and tested to create a predictive model able to assess the presence of AD traits based on SERS spectra. Specifically, we trained and tested a supervised C-SVM model that constructs a hyperplane in a high-dimensional space separating the training data into two classes. Since, in general, the larger the margin, the lower the generalization error of the classifier, a good separation is achieved by the hyperplane that has the largest distance to the nearest training data points of any class [41]*.* We selected a radial kernel and a hyperparameter C, a value proportional to the inverse of the regularization strength used during the training phase, equal to 1.0. We employed a subject-level fivefold stratified cross-validation (CV) loop to train and test the classifier. The subject-level CV divides the data between training and test sets, considering that all spectra related to the same CSF sample, and therefore to the same subject, must be entirely contained in only one of the two sets. Empirical evidence suggests that the 5- or 10-fold CV should be preferred to the leave-one-out (LOO) CV as reported by current literature and state-of-the-art machine learning development tools documentation [42, 43]. Test set data were not used during the learning process, thus preventing any form of peeking effect [44, 45]. We finally evaluated the generalization capabilities of our model on test data by computing the accuracy, specificity, sensitivity, receiver operating characteristic (ROC) curve, and area under the ROC curve (AUROC) (Additional file 1: Appendix 1).

## Results

A total number of 31 CSF samples were collected from patients with a clinical diagnosis of probable AD (*n* = 16), MCI-AD (*n* = 4) or ONC, including progressive supranuclear palsy (PSP, *n* = 1) [46], Parkinson’s disease (PD, *n* = 1) [47] and normal pressure hydrocephalus (HyC, *n* = 9) [48, 49]. Only patients with MCI-AD or AD-dementia underwent Mini-Mental State Examination (MMSE) and their scores at the time of lumbar tap are shown in Table 1. Remarkably, all MCI-AD patients converted to AD-dementia during the time between CSF collection and SAA-SERS analyses.

**Fig. 1:**
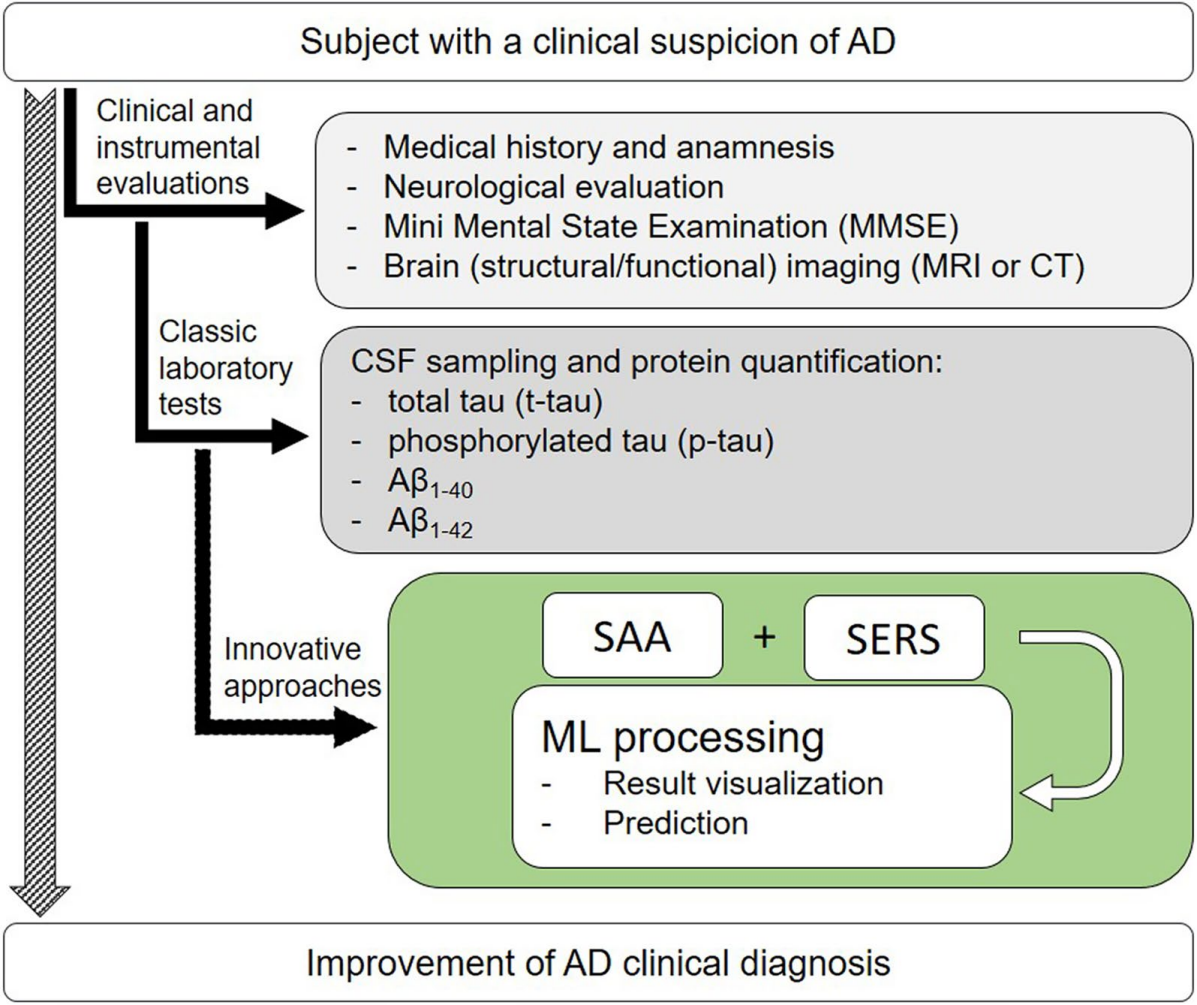
The green box highlights the level of integration of the proposed method with the classic diagnostic workup for AD. The products obtained by SAA analyses of CSF are investigated by means of SERS supported by a machine learning approach for the visualization and prediction of the results

A schematic representation which integrates our SAA-SERS approach with the conventional diagnostic work-up for AD is shown in Fig. 1. Subjects with a clinical suspicion of AD undergo several clinical and instrumental tests (light grey box, Fig. 1) and are typically subjected to CSF collection and dosage of specific protein biomarkers, including t-tau, p-tau, Aβ_1-40_ and Aβ_1-42_ (dark grey box, Fig. 1). The green box shows the integration of our combined approach in the clinical diagnostic work-up for AD which is based on the innovative analysis of CSF samples. The outcomes of this approach are finally visualized and categorized by the support of machine learning.

### CSF laboratory results

For many retrospectively collected CSF samples we had enough volume to measure the levels of specific protein markers to support the clinical diagnosis of AD, including t-tau, p-tau, Aβ_1-42_ and Aβ_1-40_. For a few cases, the amount of CSF was not enough to complete the set of biomarker analysis reported in Table 1. However, their clinical diagnosis was strongly supported by other clinical and instrumental markers. The results of CSF analysis showed that the levels of p-tau were mostly increased in AD and MCI-AD patients, while the Aβ_1-42_ levels were mainly decreased. The Aβ_1-42_/Aβ_1-40_ ratio was below 0.068 in AD and MCI-AD patients and this was indicative or predictive of AD pathology [53]. CSF analysis of ONC showed normal levels of protein markers, except for ONC#1, #6, and #7 that were characterized by a Aβ_1-42_/Aβ_1-40_ ratio below 0.068. Of note, all HyC showed low levels of Aβ_1-42_ and Aβ_1-40_ (below the normal values). This is a normal finding and is due to the fact that the increased production of CSF in these patients determines a dilution of all the proteins contained in the volume of sample analyzed.

### SAA analysis of CSF samples

**Fig. 2:**
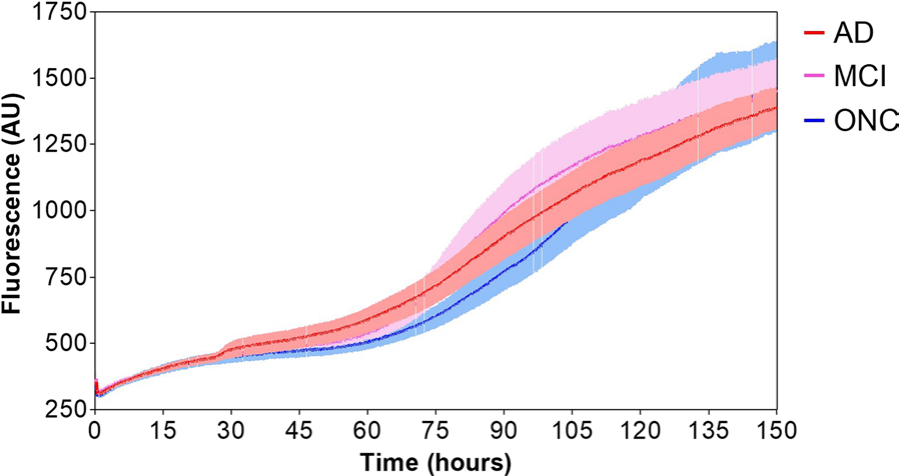
Comparison between Aβ_1-40_ aggregation kinetics triggered by AD (red line), MCI-AD (pink line) and ONC (blue line) CSF samples by SAA. The kinetics did not significantly differ between samples neither in terms of time taken to trigger Aβ_1-40_ aggregation nor in terms of fluorescence levels reached at the end of the SAA reactions

Concerning SAA analysis, we followed the protocol of Salvadores et al. [21] applying some modifications aimed at optimizing system stability and reproducibility. We observed an initial slow increase of ThT fluorescence signal (before 60 h) due to Aβ_1-40_ aggregation, followed by a more rapid aggregation kinetics either in the case of AD or ONC samples (Fig. 2). According to specific thresholds of time (75 h) and fluorescence (550 AU) we were able to determine seed-competent (seed+) and seed-incompetent (seed-) samples. In particular, we observed a seeding activity in 9/16 AD samples (AD#1, AD#2, AD#6, AD#8, AD#11, AD#12, AD#15, AD#16, AD#18), in 3/4 MCI-AD (AD#7, AD#13, AD#14) but also in 4/11 ONC (ONC#1, ONC#3, ONC#5, ONC#7, the first affected by PSP, while the others by HyC) (Additional file 1: Fig. S3), resulting in 56% (only AD) or 60% (AD + MCI-AD) sensitivity and 64% specificity in the identification of AD samples. Even by pooling together the aggregation kinetics obtained from all AD or ONC patients, we were not able at this stage to clearly distinguish AD from ONC, neither as a function of time taken to trigger Aβ_1-40_ aggregation nor of fluorescence levels reached at the end of the SAA reactions (Fig. 2).

The unsatisfactory sensitivity and specificity levels observed led us to assess the opportunity to couple SAA with SERS to improve the diagnostic performance on the basis of possible chemo-structural differences between AD and ONC products, which failed to be appreciated by SAA alone.

### Application of SERS to SAA products

**Fig. 3:**
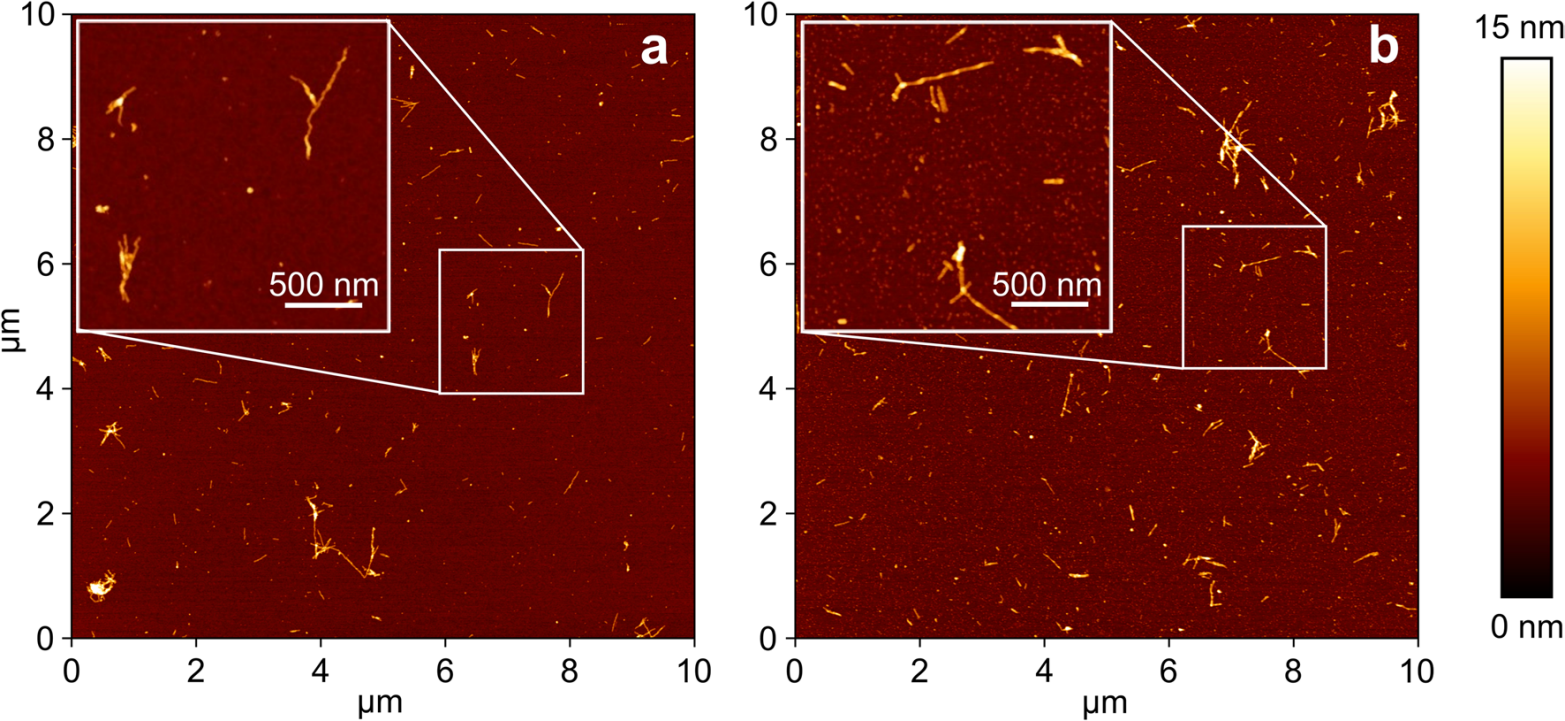
Representative AFM images of SAA products. Tapping-mode height images acquired on the SAA products generated by CSF from ONC (**a**) and AD samples (**b**), after the SAA analysis (the height bar is shown on the right). Panels show higher-resolution small-scan-size images of Αβ_1-40_ fibrils

Initially, ultrastructural characterization of the SAA end products was carried out to gain a morphological overview of the samples under scrutiny (Fig. 3). The topographical analysis by AFM highlighted the presence of fibrillary structures with a size ranging from ~ 0.1 to ~ 1.2 μm of length and ~ 6 nm of average height in all samples identified as seed-competent in the fluorescence-based SAA analysis. The formation of fibrils is in line with the high content of β-sheet structural motifs as evidenced by SAA kinetics. The widespread density of smaller globular structures revealed also an ubiquitarian sub-fibrillar content (Additional file 1: Fig. S4), characteristic of oligomeric aggregates with structural features consistent with those observed in previous studies [54, 55].

**Fig. 4:**
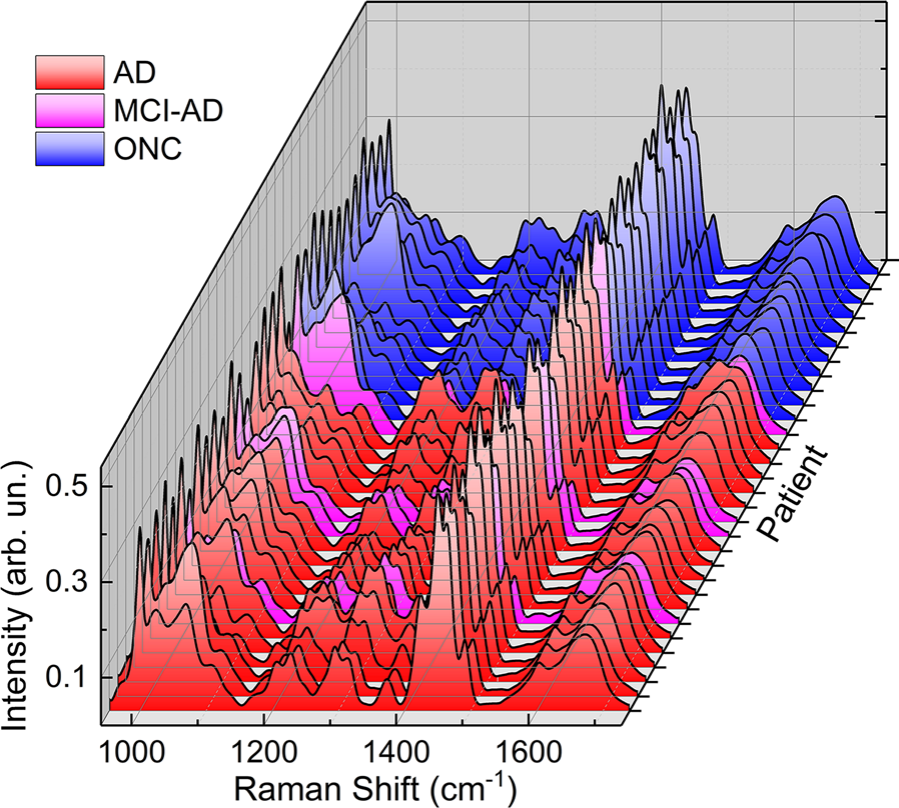
SERS spectra of SAA products of CSF samples from 16 AD, 4 MCI-AD and 11 ONC patients. For each patient sample 50 SERS spectra were acquired, elaborated (as reported in Materials and Methods section) and the average spectrum was plotted

The SERS analysis of SAA products revealed a high level of intra-sample reproducibility (Additional file 1: Figs. S5, S6), as evaluated through relative standard deviation (RSD) values ranging between 5% and 20%. This is consistent with an affordable analytical protocol, devoid of signal fluctuations due to variability in the optical response of the SERS substrate [56]. The observation of unique and reproducible shape profiles for each patient (Additional file 1: Figs. S5 and S6) suggested the existence of specific optical fingerprints identifying the different patients. More precisely, these profiles have identical vibrational bands but show a relative intensity variation (Fig. 4). These bands were identified in Additional file 1: Table S1 and ascribed to characteristic vibrational modes of Aβ_1-40_ (Additional file 1: Fig. S7), which is expected to mainly contribute to the optical response of SAA-processed samples due to its massive (micromolar) concentration in the SAA reaction mixture. The differences observed can thus reflect characteristic (in quality and quantity) interactions occurring between the oligomeric Aβ contained in the CSF and the supplemented Aβ_1-40_, during the SAA process within each CSF sample, in turn generating a characteristic SERS signature. Main bands are assigned to the aromatic tyrosine (Tyr), histidine (His), and phenylalanine (Phe) residues (1001, 1026, 1203, 1491, 1600 cm^−1^), to CC, CN and CO stretching modes (1066, 1112 cm^−1^) as well as to CH_2_ and CH_3_ deformations (1294, 1314, 1370, 1423, 1450, 1494 cm^−1^) of the peptide backbone and of the terminal amino acidic groups, and to the amide I and amide III modes at 1650–1675 and 1229 cm^−1^, respectively (Fig. S7).

### Machine learning processing

**Fig. 5:**
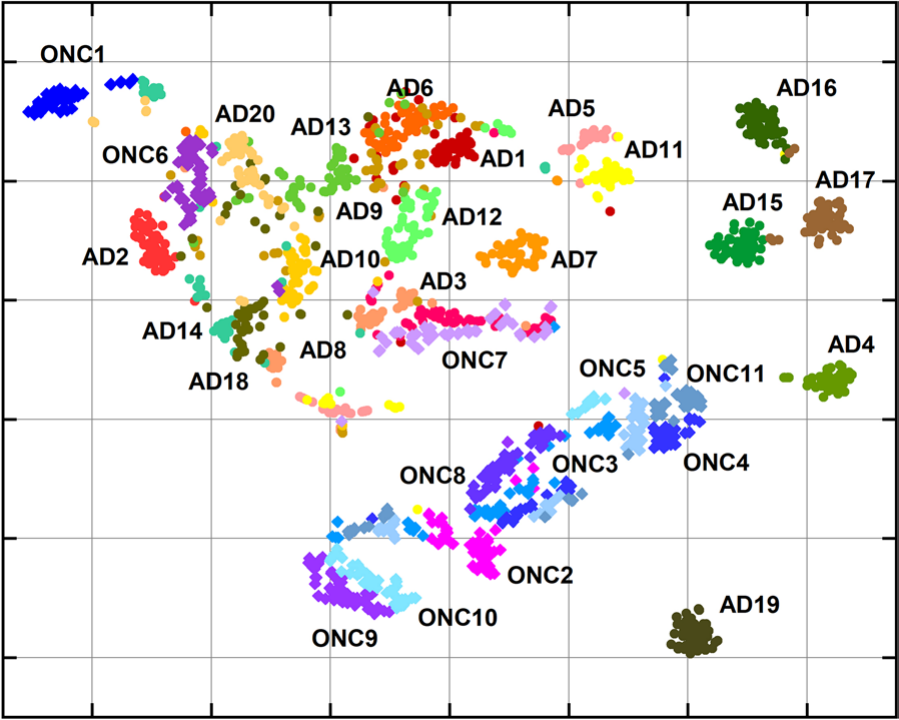
t-SNE plot obtained by applying t-SNE algorithm to SERS spectra of SAA products of CSF samples. Circles in red-yellow-green color scale refer to SERS spectra acquired from AD patients, while blue-violet diamonds point out to ONC patients. Same colored group symbol refers to the same patient labeled by a number following the list in Table 1

To manage our dataset collected by SERS analysis, we adopted a machine learning approach to differentiate and classify the spectral data. We analyzed SERS spectra using the unsupervised t-SNE algorithm to get a bi-dimensional view of spectra distribution across all patients in the study [40]. Indeed, t-SNE is a widely used method in machine learning, allowing the exploration of high-dimensional data thanks to comprehensive two-dimensional maps. Graphically, we found that t-SNE led to a clean clustering of the samples belonging to patients with a clinical diagnosis of AD mostly separated from those of ONC (Fig. 5). Therefore, after SAA running, AD and ONC samples were mostly separated in two distinct groups at a first glance. However, a few ONC samples (ONC#1, ONC#6 and ONC#7, associated to PSP and HyC, respectively) clustered with the AD group and vice-versa (AD#4 and AD#19), denoting a deviation from the common trend, which requires further evaluation after cross-referencing with clinical data. Remarkably, ONC samples clustering in the group of AD (ONC#1, ONC#6, ONC#7) showed an Aβ_1-42_/Aβ_1-40_ ratio in the range of AD pathology (Table 1), which might well justify their AD-like behavior in the t-SNE plot. In addition, ONC#1 and ONC#7 were also able to trigger Aβ_1-40_ aggregation by SAA, while ONC#6 did not. On the other hand, conclusions on unexpected responses of AD#4 and AD#19 cannot be immediately drawn based on the available clinical, neuropsychological, and instrumental data.

Interestingly, all MCI-AD cases (AD#7, AD#13, AD#14 and AD#20) clustered together with the other AD samples (Fig. 5). On the opposite, it is worth noting the lack of any noticeable differentiation in the case of samples unprocessed by SAA (Additional file 1: Fig. S8), indicating that the SAA aggregation step is essential for sample discrimination.

**Fig. 6:**
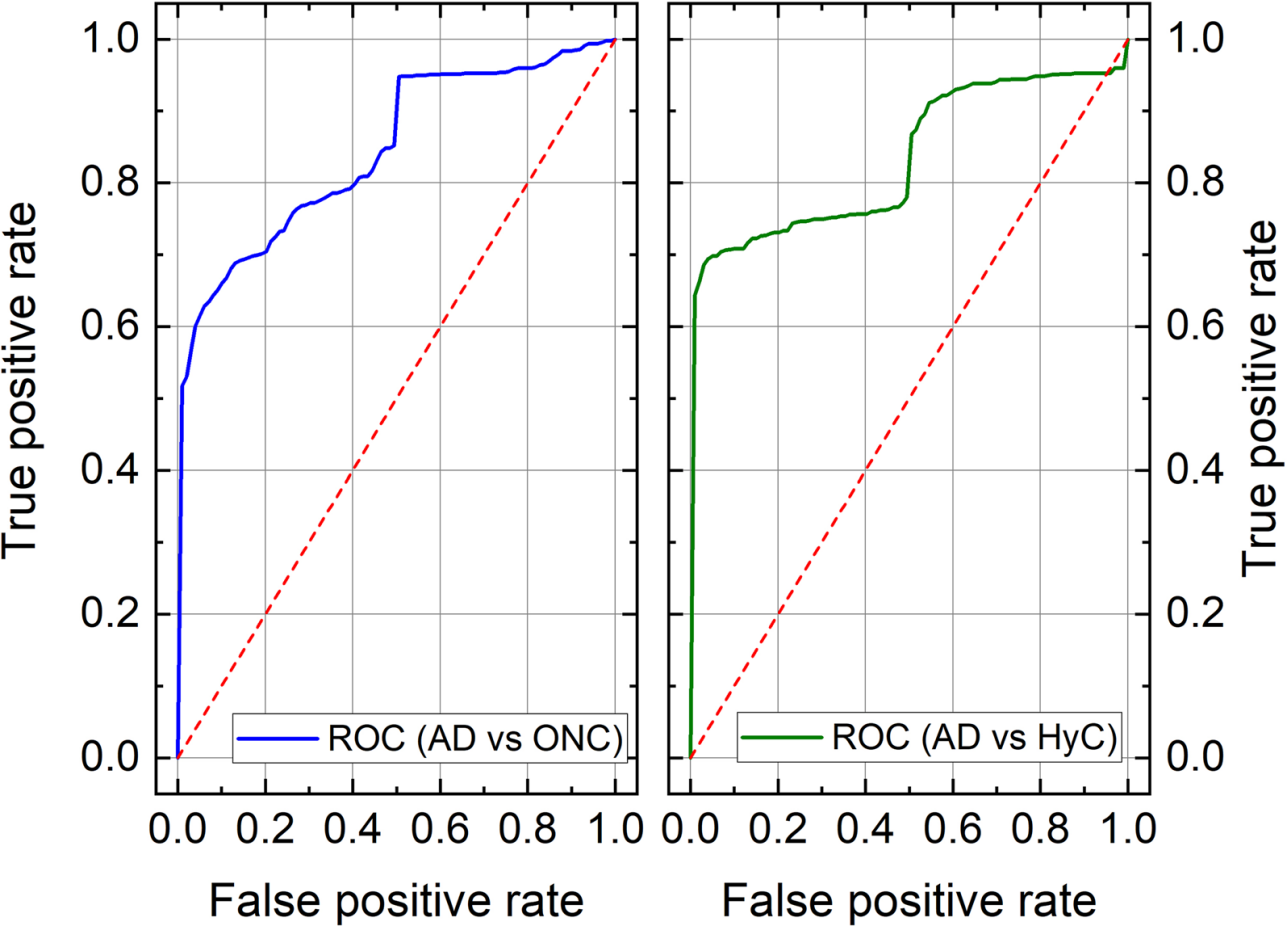
Average ROC curve (over the fivefold CV) in the case of AD patients versus ONC patients analysis (blue line, left panel) and in the case of AD patients versus HyC patients (green line, right panel)

As a further step in the discrimination of AD patients, we set up a predictive model able to assess the presence of AD traits based on SERS spectra. Specifically, we performed two separate analyses. In the first one, we aimed to differentiate AD patients from all ONC; in the second one, we removed non-HyC patients (ONC#1 and ONC#2 in Table 1, affected by PSP and PD, respectively) from the ONC group to compare AD patients with HyC patients only. Quantitatively, the classification performances were good in both analyses. We reported the mean values (over the 5-fold CV) of AUROC, accuracy, sensitivity, and specificity in the training and the test sets in Additional file 1: Table S2 and Table 2, respectively. Specifically, in both test sets, AUROC values were above 0.8, with sensitivity and specificity greater than 0.8 and 0.7, respectively. The goodness of the models obtained in both analyses can also be seen in the graphs of the ROC curves (Fig. 6).

**Table 2:** Performance of the classifier in the test set for both AD patients versus ONC patients and AD versus HyC

Dataset	AUROC	Accuracy	Sensitivity	Specificity
AD vs ONC	0.85 (0.16)	0.84 (0.12)	0.88 (0.13)	0.77 (0.22)
AD vs HyC	0.84 (0.20)	0.85 (0.13)	0.89 (0.13)	0.81 (0.20)

The HyC group was a subgroup of ONC. Data are mean (standard deviation) over the iterations of the 5-fold cross-validation. AD: Alzheimer’s disease; ONC: other neurodegenerative conditions; AUROC: area under the receiver operating characteristic curve; HyC: patients with normal pressure hydrocephalus

The comprehensive set of results shown above supports that  our approach can discriminate CSF of AD and MCI-AD patients from CSF of patients with ONC, with some exceptions as occurred in the case of abnormal AD-like dosages in the CSF of ONC patients. We did not identify specific clinical features (e.g. atypical clinical features), which might explain why AD#15, AD#16, and AD#17 clustered together and separated from the other AD samples after applying the t-SNE algorithm to the SERS spectra of SAA final products (Fig. 5).

## Discussion

The clinical diagnosis of AD is based on clinical assessment as well as instrumental and laboratory findings, which include the measurement of several CSF biomarkers, including Aβ_1-42_, Aβ_1-40_, t-tau, and p-tau. Molecular imaging tools can also highlight abnormal Aβ accumulation in the brain [57].

Although the clinical diagnostic criteria for AD enable accurate disease identification, they have some limitations, including the lack of early, sensitive and specific tests to recognize patients in their early disease stages using easily accessible tissues. Currently, the analysis of CSF biomarkers is of utmost importance for supporting an AD diagnosis. However, there is a lack of CSF markers that can accurately differentiate AD from other dementias and eventually allowing the recognition of different disease phenotypes, especially in the early disease stages. Even with the development of highly sensitive technologies, including the single-molecule array and other mass spectrometry techniques which provide important opportunities for the development of blood-based biomarkers for AD, no disease-specific markers have been discovered yet [58–63]. It is well accepted that Aβ oligomers play a crucial role in AD pathogenesis and recent evidence shows that they can circulate in the CSF, thus representing specific markers for AD [5, 21, 64]. In 2014, it was demonstrated that seeding-competent Aβ oligomers were detectable by SAA analysis in the CSF of patients with a diagnosis of probable AD [21]. In the current study, we adapted the SAA technology for the analysis of CSF collected from extensively-characterized patients with a diagnosis of probable AD, MCI-AD and other neurological conditions, including PSP, PD and HyC. Compared to the study by Salvadores et al. [21], we used monomers of Aβ_1-40_ peptide as reaction substrate since they could be easily handled and their aggregation could be efficiently triggered by minute amounts of Aβ_1-42_ contained in the brains of patients with AD, in turn improving the stability of the system. However, our SAA assay was able to identify seeding-competent oligomers only in 60% of the total AD and MCI-AD CSF samples with an unsatisfactorily specificity of 64%. To improve the performance of our analytical approach, we subjected the SAA end-products to SERS analysis and this strategy enabled us to discriminate between AD patients and ONC, achieving a sensitivity of 88% and a specificity of 77%. Interestingly, all samples collected from patients diagnosed with MCI-AD at the time of CSF withdrawal, clustered together with the group of AD dementia. These results suggest that SAA-SERS can potentially recognize AD pathology in the early disease stage. Furthermore, among the group of AD patients, 3 CSF samples (AD#15, AD#16, AD#17) clustered apart from the others, which may reflect the phenotypical heterogeneity of the disease. Notably, when simply considering the SAA aggregation kinetics, the CSF samples from AD#15 and AD#16 were able to seed Aβ_1-40_ while that of AD#17 did not. Thus, we did not find any possible link between SAA aggregation kinetics and SERS findings.

One of the most interesting findings is that 3 ONC (#1, #6, #7, the first affected by PSP, the others by HyC) clustered together with the AD by SERS analysis and they were characterized by a CSF Aβ_1-42_/Aβ_1-40_ ratio suggestive of AD pathology. Thus, we might not exclude a coincidental AD pathology in these patients. Interestingly, two of these samples (ONC#1 and ONC#7) were also capable of efficiently triggering Aβ_1-40_ aggregation by SAA_._ Therefore, we might not exclude the possibility of the presence of seeding-competent Aβ oligomers in these ONC CSF, which are not associated with AD pathology but still capable of seeding Aβ_1-40_ aggregation. These results support the fact that different strains of Aβ are responsible for the clinical variability of AD and in some cases they might be present in tissues of patients with other neurological conditions, thus making the clinical diagnosis of AD even more challenging [17, 65, 66]. Although the number of AD samples included in the study was limited due to the criteria for including only extensively characterized CSF/patients, these findings suggest that seeding-competent Aβ oligomers are detectable in the CSF of AD patients and that the integration of SAA with SERS makes it possible for clinical diagnosis of AD since the earliest disease stages. Considering the proof-of-concept nature of this work, it will be important to perform additional studies in larger populations of AD patients and controls to verify the accuracy of our approach. It should be highlighted that our approach can recognize the peripheral effects of Aβ pathology occurring in the brain, but this alteration does not only characterize AD and can be observed as a coincidental finding in a few other neurodegenerative conditions (e.g. dementia with Lewy bodies and frontotemporal dementia syndromes) [67, 68]. One of the weaknesses of the study was that none of the patients underwent autopsy and our findings could be compared only with the clinical/instrumental assessment of these patients.

## Conclusions

In conclusion, the results of our amplification-based approach must be well interpreted and contextualized in the clinical setting in which it is applied. Herein, we describe a proof-of-concept study in which the unique features of two techniques are successfully combined to improve Aβ-oligomer detection and characterization in the CSF of patients with a clinical diagnosis of AD. Our findings suggest that this approach might help recognize or even predict disease features. Stratifying AD patients, especially in their early disease stage, would maximize the efficacy of therapeutic treatment, especially considering that anti-amyloid drugs (e.g., Aducanumab and Lecanemab) are believed to be effective only when administered at the very early stages of the disease, and that their efficacy might depend on disease phenotypes [69–72]. Finally, it would be important to perform longitudinal studies using CSF periodically collected from the same patients to evaluate whether our findings correlate with disease stage and progression. However, this is practically unfeasible and other biological tissues such as blood, urine and olfactory mucosa should be investigated for the presence of pathological Aβ oligomers using the new SAA-SERS approach.

## Supplementary Information

**Additional file 1**. **Fig. S1** Biochemical analyses of Aβ_1-40_ peptide used as a SAA reaction substrate and picture of a AgNWs@PTFE substrate used to study SAA products. **Fig. S2** Comparison between raw (top panel) and post-processed (bottom panel) SERS spectra acquired from the SAA products of CSF of an AD patient. **Fig. S3** Seed amplification assay of CSF samples collected from AD-dementia, MCI-AD (top panel) and ONC (bottom panel). **Fig. S4** AFM imaging. **Fig. S5** SERS spectra of SAA products of CSF samples from 16 AD and 4 MCI-AD patients. **Fig. S6** SERS spectra of SAA products of CSF samples from 11 ONC patients. **Fig. S7** SERS spectrum of Aβ_1-40_ aggregates after SAA (blue line) as compared to spectra of SAA products from an AD (red line) and a ONC patient sample (green line). **Fig. S8**. t-SNE plot obtained by applying t-SNE algorithm to SERS spectra of CSF samples before undergoing seed amplification. **Appendix 1**–Machine Learning. **Table S1** Assignment of SERS bands. **Table S2** Performances of the classifier in training set for both the machine learning analyses, i.e., AD patients versus ONC (non-AD patients), and AD patients versus HyC patients.

## Data Availability

All data associated with this study are contained in the paper or in the supplementary materials. Scripts for statistical analysis can be found in the following libraries: https://scikit-learn.org/stable/modules/generated/sklearn.manifold.TSNE.html, https://scikit-learn.org/stable/modules/generated/sklearn.svm.SVC.html?highlight=svc#sklearn.svm.SVC.

## Abbreviations

AD: Alzheimer's disease
SAA: Seed amplification assay
SERS: Surface-enhanced Raman spectroscopy
MCI-AD: Mild-cognitive impairment due to AD
CSF: Cerebrospinal fluid
ThT: Thioflavin-T
ONC: Other neurological conditions
AFM: Atomic force microcopy
AgNW: Silver nanowire
CV: Cross-validation
ROC: Receiver operating characteristic
AUROC: Area under receiver operating characteristic curve
PSP: Progressive supranuclear palsy
PD: Parkinson’s disease
HyC: Normal pressure hydrocephalus
MMSE: Mini-mental state examination
t-SNE: T-distributed stochastic neighbor embedding

## References

[1] International D, Patterson C. World Alzheimer Report 2018 - The state of the art of dementia research: New frontiers. Alzheimer’s Dis Int. 2018.

[2] Ballard C, Gauthier S, Corbett A, Brayne C, Aarsland D, Jones E (2011). Alzheimer’s disease. Lancet.

[3] Thal DR, Fändrich M (2015). Protein aggregation in Alzheimer’s disease: Aβ and τ and their potential roles in the pathogenesis of AD. Acta Neuropathol.

[4] Carreiras M, Mendes E, Perry M, Francisco A, Marco-Contelles J (2013). The multifactorial nature of Alzheimer's disease for developing potential therapeutics. Curr Top Med Chem.

[5] Hardy JA, Higgins GA (1992). Alzheimer’s disease: the amyloid cascade hypothesis. Science.

[6] Selkoe DJ, Hardy J (2016). The amyloid hypothesis of Alzheimer’s disease at 25 years. EMBO Mol Med.

[7] Selkoe DJ (1989). Amyloid β protein precursor and the pathogenesis of Alzheimer’s disease. Cell.

[8] Bloom GS (2014). Amyloid-β and Tau. JAMA Neurol.

[9] DeKosky ST, Scheff SW (1990). Synapse loss in frontal cortex biopsies in Alzheimer’s disease: correlation with cognitive severity. Ann Neurol.

[10] Crews L, Masliah E (2010). Molecular mechanisms of neurodegeneration in Alzheimer’s disease. Hum Mol Genet.

[11] Frisoni GB, Altomare D, Thal DR, Ribaldi F, van der Kant R, Ossenkoppele R (2022). The probabilistic model of Alzheimer disease: the amyloid hypothesis revised. Nat Rev Neurosci.

[12] Rasmussen J, Mahler J, Beschorner N, Kaeser SA, Häsler LM, Baumann F (2017). Amyloid polymorphisms constitute distinct clouds of conformational variants in different etiological subtypes of Alzheimer’s disease. Proc Natl Acad Sci.

[13] Condello C, Lemmin T, Stöhr J, Nick M, Wu Y, Maxwell AM (2018). Structural heterogeneity and intersubject variability of Aβ in familial and sporadic Alzheimer’s disease. Proc Natl Acad Sci.

[14] Condello C, Stöehr J (2018). Aβ propagation and strains: implications for the phenotypic diversity in Alzheimer’s disease. Neurobiol Dis.

[15] Qiang W, Yau W-M, Lu J-X, Collinge J, Tycko R (2017). Structural variation in amyloid-β fibrils from Alzheimer’s disease clinical subtypes. Nature.

[16] Stohr J, Condello C, Watts JC, Bloch L, Oehler A, Nick M (2014). Distinct synthetic A prion strains producing different amyloid deposits in bigenic mice. Proc Natl Acad Sci.

[17] Di Fede G, Catania M, Maderna E, Ghidoni R, Benussi L, Tonoli E (2018). Molecular subtypes of Alzheimer’s disease. Sci Rep.

[18] McKhann GM, Knopman DS, Chertkow H, Hyman BT, Jack CR, Kawas CH (2011). The diagnosis of dementia due to Alzheimer’s disease: recommendations from the National Institute on aging-Alzheimer’s association workgroups on diagnostic guidelines for Alzheimer’s disease. Alzheimer’s Dement.

[19] Albert MS, DeKosky ST, Dickson D, Dubois B, Feldman HH, Fox NC (2011). The diagnosis of mild cognitive impairment due to Alzheimer’s disease: recommendations from the national institute on aging-Alzheimer’s association workgroups on diagnostic guidelines for Alzheimer’s disease. Alzheimers Dement.

[20] Bistaffa E, Tagliavini F, Matteini P, Moda F (2020). Contributions of molecular and optical techniques to the clinical diagnosis of Alzheimer’s disease. Brain Sci.

[21] Salvadores N, Shahnawaz M, Scarpini E, Tagliavini F, Soto C (2014). Detection of misfolded Aβ oligomers for sensitive biochemical diagnosis of Alzheimer’s disease. Cell Rep.

[22] Ji M, Arbel M, Zhang L, Freudiger CW, Hou SS, Lin D (2018). Label-free imaging of amyloid plaques in Alzheimer’s disease with stimulated raman scattering microscopy. Sci Adv.

[23] Lomont JP, Rich KL, Maj M, Ho JJ, Ostrander JS, Zanni MT (2018). Spectroscopic signature for stable β-amyloid fibrils versus β-sheet-rich oligomers. J Phys Chem B.

[24] Zikic B, Bremner A, Talaga D, Lecomte S, Bonhommeau S (2021). Tip-enhanced Raman spectroscopy of Aβ(1–42) fibrils. Chem Phys Lett.

[25] Flynn JD, Lee JC (2018). Raman fingerprints of amyloid structures. Chem Commun.

[26] Abedin F, Kandel N, Tatulian SA (2021). Effects of Aβ-derived peptide fragments on fibrillogenesis of Aβ. Sci Rep.

[27] Plasmonics in Chemistry and Biology. Jenny Stanford Publ. Available from: https://www.jennystanford.com/9789814800037/plasmonics-in-chemistry-and-biology.

[28] Applications of Raman Spectroscopy to Biology. IOS Press. Available from: https://www.jospress.com/catalog/books/applications-of-raman-specroscopy-to-biology.

[29] Bruzas I, Lum W, Gorunmez Z, Sagle L (2018). Advances in surface-enhanced Raman spectroscopy (SERS) substrates for lipid and protein characterization: sensing and beyond. Analyst.

[30] Cialla-May D, Zheng X-S, Weber K, Popp J (2017). Recent progress in surface-enhanced Raman spectroscopy for biological and biomedical applications: from cells to clinics. Chem Soc Rev.

[31] Polykretis P, Banchelli M, D’Andrea C, de Angelis M, Matteini P (2022). Raman spectroscopy techniques for the investigation and diagnosis of Alzheimer’s disease. Front Biosci.

[32] D’Andrea C, Foti A, Cottat M, Banchelli M, Capitini C, Barreca F (2018). Nanoscale discrimination between toxic and nontoxic protein misfolded oligomers with tip-enhanced Raman spectroscopy. Small.

[33] Banchelli M, Cascella R, D’Andrea C, Cabaj L, Osticioli I, Ciofini D (2020). Nanoscopic insights into the surface conformation of neurotoxic amyloid β oligomers. RSC Adv.

[34] De Luca CMG, Consonni A, Cazzaniga FA, Bistaffa E, Bufano G, Quitarrini G (2021). The alpha-synuclein RT-QuIC products generated by the olfactory mucosa of patients with Parkinson’s disease and multiple system atrophy induce inflammatory responses in SH-SY5Y Cells. Cells.

[35] Leitão MJ, Silva-Spínola A, Santana I, Olmedo V, Nadal A, Le Bastard N (2019). Clinical validation of the Lumipulse G cerebrospinal fluid assays for routine diagnosis of Alzheimer’s disease. Alzheimer’s Res Ther.

[36] Gobom J, Parnetti L, Rosa-Neto P, Vyhnalek M, Gauthier S, Cataldi S (2022). Validation of the LUMIPULSE automated immunoassay for the measurement of core AD biomarkers in cerebrospinal fluid. Clin Chem Lab Med.

[37] Banchelli M, Amicucci C, Ruggiero E, D’Andrea C, Cottat M, Ciofini D (2019). Spot-on SERS detection of biomolecules with laser-patterned dot arrays of assembled silver nanowires. ChemNanoMat.

[38] Barucci A, D’Andrea C, Farnesi E, Banchelli M, Amicucci C, De Angelis M (2021). Label-free SERS detection of proteins based on machine learning classification of chemo-structural determinants. Analyst.

[39] Krafft C, Schmitt M, Schie IW, Cialla-May D, Matthäus C, Bocklitz T (2017). Label-free molecular imaging of biological cells and tissues by linear and nonlinear raman spectroscopic approaches. Angew Chem Int Ed Engl.

[40] van der Maaten L, Hinton G (2008). Visualizing data using t-SNE. J Mach Learn Res.

[41] Pattern recognition and machine learning. Available from: https://link.springer.com/book/9780387310732.

[42] Breiman L, Spector P (1992). Submodel selection and evaluation in regression. The X-Random case Int Stat Rev.

[43] Hastie T, Tibshirani R, Friedman J (2009). The Elements of Statistical Learning: Data Mining, Inference, Prediction.

[44] Diciotti S, Ciulli S, Mascalchi M, Giannelli M, Toschi N (2013). The, “Peeking” effect in supervised feature selection on diffusion tensor imaging data. Am J Neuroradiol.

[45] Yagis E, Atnafu SW, Seco G, de Herrera A, Marzi C, Scheda R, Giannelli M (2021). Effect of data leakage in brain MRI classification using 2D convolutional neural networks. Sci Rep.

[46] Höglinger GU, Respondek G, Stamelou M, Kurz C, Josephs KA, Lang AE (2017). Clinical diagnosis of progressive supranuclear palsy: the movement disorder society criteria. Mov Disord.

[47] Postuma RB, Poewe W, Litvan I, Lewis S, Lang AE, Halliday G (2018). Validation of the MDS clinical diagnostic criteria for Parkinson’s disease. Mov Disord.

[48] Williams MA, Malm J (2016). Diagnosis and treatment of idiopathic normal pressure hydrocephalus. Contin Lifelong Learn Neurol.

[49] Relkin N, Marmarou A, Klinge P, Bergsneider M, McL BP (2005). Diagnosing idiopathic normal-pressure hydrocephalus. Neurosurgery.

[50] Vanderstichele H, De Vreese K, Blennow K, Andreasen N, Sindic C, Ivanoiu A (2006). Analytical performance and clinical utility of the INNOTEST® PHOSPHO-TAU(181P) assay for discrimination between Alzheimer’s disease and dementia with Lewy bodies. Clin Chem Lab Med.

[51] Sjögren M, Vanderstichele H, Ågren H, Zachrisson O, Edsbagge M, Wikkelsø C (2001). Tau and Aβ42 in cerebrospinal fluid from healthy adults 21–93 years of age: establishment of reference values. Clin Chem.

[52] Bellomo G, Indaco A, Chiasserini D, Maderna E, Paolini Paoletti F, Gaetani L (2021). Machine learning driven profiling of cerebrospinal fluid core biomarkers in Alzheimer’s disease and other neurological disorders. Front Neurosci.

[53] Doecke G, Rembach A, Villemagne D, Varghese E, Rainey-Smith F, Sarros L (2017). Concordance between cerebrospinal fluid biomarkers with Alzheimer’s disease pathology between three independent assay platforms. J Alzheimers Dis..

[54] Moores G, Drolle A, Attwood D, Simons E, Leonenko F (2011). Effect of surfaces on amyloid fibril formation. PLoS One..

[55] Blackley HKL, Patel N, Davies MC, Roberts CJ, Tendler SJB, Wilkinson MJ (1999). Morphological development of β(1–40) amyloid fibrils. Exp Neurol.

[56] Pérez-Jiménez AI, Lyu D, Lu Z, Liu G, Ren B (2020). Surface-enhanced Raman spectroscopy: benefits, trade-offs and future developments. Chem Sci.

[57] Mallik A, Drzezga A, Minoshima S (2017). Clinical amyloid imaging. Semin Nucl Med.

[58] Rissin DM, Kan CW, Campbell TG, Howes SC, Fournier DR, Song L (2010). Single-molecule enzyme-linked immunosorbent assay detects serum proteins at subfemtomolar concentrations. Nat Biotechnol.

[59] Ovod V, Ramsey KN, Mawuenyega KG, Bollinger JG, Hicks T, Schneider T (2017). Amyloid β concentrations and stable isotope labeling kinetics of human plasma specific to central nervous system amyloidosis. Alzheimers Dement.

[60] Nakamura A, Kaneko N, Villemagne VL, Kato T, Doecke J, Doré V (2018). High performance plasma amyloid-β biomarkers for Alzheimer’s disease. Nature.

[61] Yang SY, Chiu MJ, Chen TF, Horng HE (2017). Detection of plasma biomarkers using immunomagnetic reduction: a promising method for the early diagnosis of Alzheimer’s disease. Neurol Ther.

[62] Kim Y, Yoo YK, Kim HY, Roh JH, Kim J, Baek S (2019). Comparative analyses of plasma amyloid-β levels in heterogeneous and monomerized states by interdigitated microelectrode sensor system. Sci Adv.

[63] Li D, Mielke MM (2019). An update on blood-based markers of Alzheimer’s disease using the SiMoA platform. Neurol Ther.

[64] Walsh DM, Selkoe DJ (2007). A beta oligomers - a decade of discovery. J Neurochem.

[65] Cohen M, Appleby B, Safar JG (2016). Distinct prion-like strains of amyloid beta implicated in phenotypic diversity of Alzheimer’s disease. Prion.

[66] Makowski L (2020). The structural basis of amyloid strains in Alzheimer’s disease. ACS Biomater Sci Eng.

[67] Armstrong RA, Cairns NJ, Lantos PL (2000). Beta-amyloid deposition in the temporal lobe of patients with dementia with lewy bodies: comparison with non-demented cases and Alzheimer’s disease. Dement Geriatr Cogn Disord.

[68] Tan RH, Kril JJ, Yang Y, Tom N, Hodges JR, Villemagne VL (2017). Assessment of amyloid β in pathologically confirmed frontotemporal dementia syndromes. Alzheimer’s Dement Diagnosis Assess Dis Monit.

[69] Alexander GC, Emerson S, Kesselheim AS (2021). Evaluation of Aducanumab for Alzheimer disease. JAMA.

[70] Tagliavini F, Tiraboschi P, Federico A (2021). Alzheimer’s disease: the controversial approval of Aducanumab. Neurol Sci.

[71] Padovani A, Caratozzolo S, Rozzini L, Pilotto A, Benussi A, Tedeschi G (2022). “Real-world” eligibility for aducanumab depends on clinical setting and patients’ journey. J Am Geriatr Soc.

[72] Söderberg L, Johannesson M, Nygren P, Laudon H, Eriksson F, Osswald G (2022). Lecanemab, Aducanumab, and Gantenerumab — binding profiles to different forms of amyloid-beta might explain efficacy and side effects in clinical trials for Alzheimer’s disease. Neurotherapeutics.

