# Supplementary material for: Impact of seed amplification assay and surface-enhanced Raman spectroscopy combined approach on the clinical diagnosis of Alzheimer’s disease
Source: Transl Neurodegener. 2023 Jul 12;12:35. doi: 10.1186/s40035-023-00367-9 (PMC10337059; doi:10.1186/s40035-023-00367-9)
Supplement: Supplementary file 1 — Additional file 1. Fig. S1 Biochemical analyses of Aβ1-40 peptide used as a SAA reaction substrate and picture of a AgNWs@PTFE substrate used to study SAA products. Fig. S2 Comparison between raw (top panel) and post-processed (bottom panel) SERS spectra acquired from the SAA products of CSF of an AD patient. Fig. S3 Seed amplification assay of CSF samples collected from AD-dementia, MCI-AD (top panel) and ONC (bottom panel). Fig. S4 AFM imaging. Fig. S5 SERS spectra of SAA products of CSF samples from 16 AD and 4 MCI-AD patients. Fig. S6 SERS spectra of SAA products of CSF samples from 11 ONC patients. Fig. S7 SERS spectrum of Aβ1-40 aggregates after SAA (blue line) as compared to spectra of SAA products from an AD (red line) and a ONC patient sample (green line). Fig. S8. t-SNE plot obtained by applying t-SNE algorithm to SERS spectra of CSF samples before undergoing seed amplification. Appendix 1–Machine Learning. Table S1 Assignment of SERS bands. Table S2 Performances of the classifier in training set for both the machine learning analyses, i.e., AD patients versus ONC (non-AD patients), and AD patients versus HyC patients. [file 40035_2023_367_MOESM1_ESM.docx]

**Additional file 1**

**
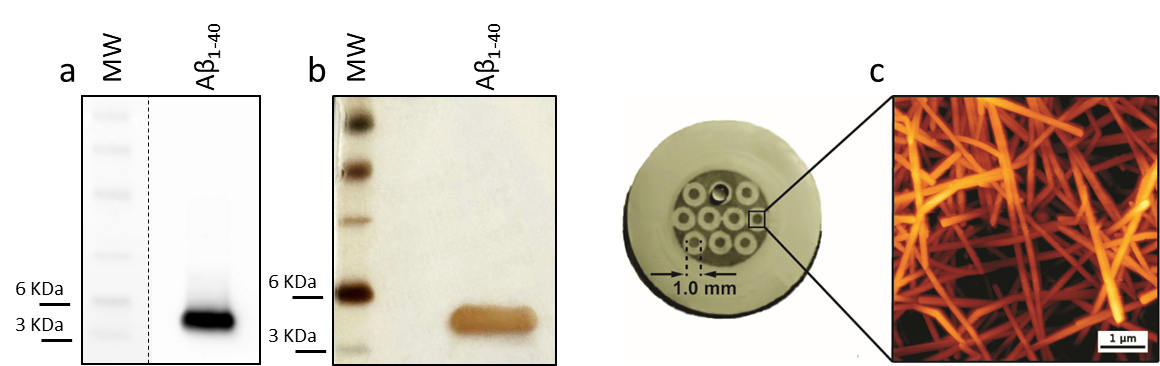
**

**Figure S1.** Biochemical analyses of Aβ_1-40_ peptide used as a SAA reaction substrate and picture of a AgNWs@PTFE substrate used to study SAA products. a) Western blot of Aβ_1-40_ peptide after its dissolution in NaOH 10mM. The membrane was probed with the 6E10 monoclonal antibody, showing the monomeric form of the peptide migrating at 4kDa. Dashed lines indicate cropped images. b) Silver stain analysis of Aβ_1-40_ peptide after its dissolution in NaOH 10mM. Pierce silver stain kit (Thermo scientific) was used and shows the presence of a band corresponding to the monomeric form of the peptide, also migrating at 4kDa. Molecular weight markers are reported on the left side of the Western blot and silver staining images. c) Representative picture of a AgNWs@PTFE substrate including 10-patterned silver spots. Each spot measures 1 mm in diameter and allows the deposition of SAA products. An AFM topography image of the AgNWs layer is reported on the right. AgNWs with length ranging from a few to tens of µm and a diameter of 80 nm form a network with multiple intersections behaving as efficient SERS hotspots under a visible/NIR laser excitation wavelength [1,2]

**Figure S2.** Comparison between raw (top panel) and post-processed (bottom panel) SERS spectra acquired from the SAA products of CSF of an AD patient. Processing consisted in a sequence of mathematical functions [2,3], in which the spectra were corrected for cosmic ray spikes, baselined (polynomial fit), smoothed and area normalized by using Labspec 6 software (Horiba, France).

**
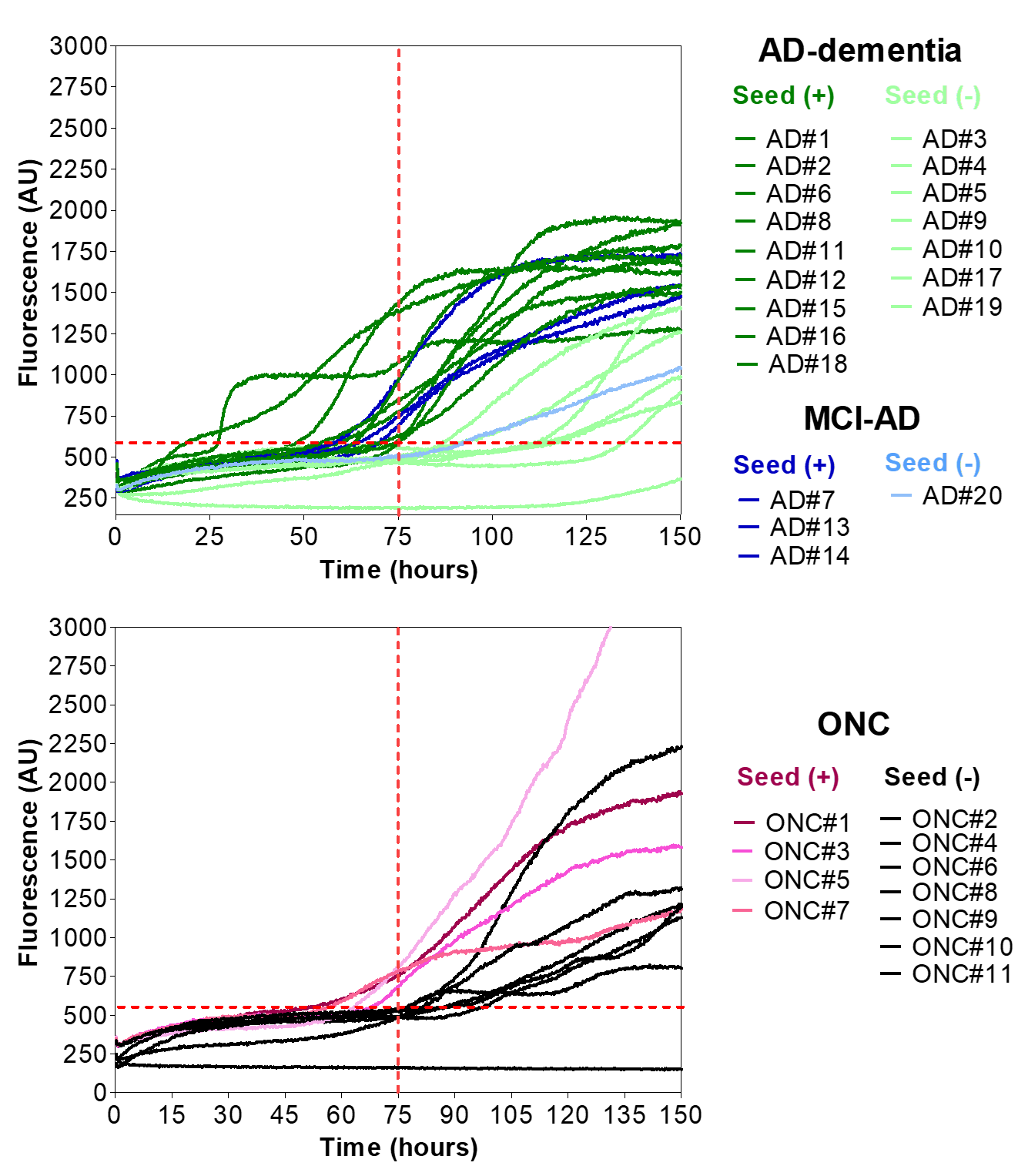
**

**Figure S3.** Seed amplification assay of CSF samples collected from AD-dementia, MCI-AD (top panel) and ONC (bottom panel). A seeding activity is observed in 9/16 AD-dementia samples (AD#1, AD#2, AD#6, AD#8, AD#11, AD#12, AD#15, AD#16, AD#18), in 3/4 MCI-AD (AD#7, AD#13, AD#14) but also in 4/11 ONC (#1, #3, #5, #7).


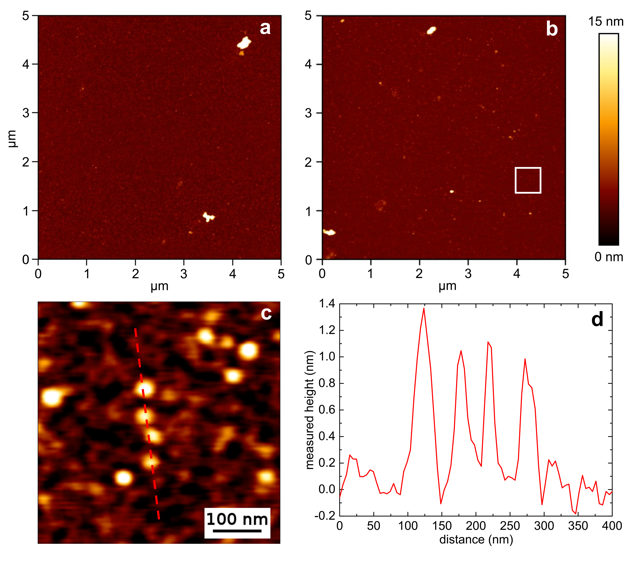


**Figure S4.** AFM imaging. Tapping-mode height images acquired on the CSF of a ONC (a) and an AD sample (b), before the SAA treatment (the height bar is shown on the right). (c) Higher resolution magnification of the white panel highlighted in figure (b), showing Αβ oligomers. The red dotted line represents the axis along which the height profile of the Αβ oligomers shown in (d) has been measured. Correcting the measured widths for the tip-induced broadening in the image plane as previously reported [4], the estimated diameter of the oligomeric aggregates is ~30 nm, in accordance with previous studies [5,6].

**Figure S5**. SERS spectra of SAA products of CSF samples from 16 AD and 4 MCI-AD patients. For each sample 50 SERS spectra were acquired, elaborated (as reported in Materials and Methods section) and the average spectrum and the standard deviation were plotted (black line and red area, respectively)

**Figure S6**. SERS spectra of SAA products of CSF samples from 11 ONC patients. For each sample 50 SERS spectra were acquired, elaborated (as reported in Materials and Methods section) and the average spectrum and the standard deviation were plotted (black line and red area, respectively).

**Figure S7**. SERS spectrum of Aβ_1−40_ aggregates after SAA (blue line) as compared to spectra of SAA products from an AD (red line) and a ONC patient sample (green line). Main Raman vibrational modes are highlighted by vertical dashed lines and assigned in Table S1. Each spectrum is the average of 50 acquisition.

**Figure S8**. t-SNE plot obtained by applying t-SNE algorithm to SERS spectra of CSF samples before undergoing seed amplification

**Appendix 1**

**Machine learning**

t-SNE parameters:

par_perplexity = 20

exageration = 12

Library: https://scikit-learn.org/stable/modules/generated/sklearn.manifold.TSNE.html

SVM parameters:

svm.SVC(C=1.0, kernel='rbf', degree=3, gamma='scale', coef0=0.0, shrinking=True, probability=True, tol=0.001, cache_size=200, class_weight=None, verbose=False, max_iter=- 1, decision_function_shape='ovr', break_ties=False, random_state=42)

Library: https://scikit-learn.org/stable/modules/generated/sklearn.svm.SVC.html?highlight=svc#sklearn.svm.SVC

**Table S1.** Assignment of SERS bands

| Raman shift (cm^-1^) | Assignment | Reference |
| --- | --- | --- |
| 1001 | Phe | Podstawka et al., 2004 [7] |
| 1026 | Phe | Podstawka et al., 2004 [7] |
| 1066 | νCC, νCN, νCO | Roy et al., 2017 [8] |
| 1112 | νCC | Roy et al., 2017 [8] |
| 1203 | Tyr, Phe | Podstawka et al., 2004 [7] |
| 1229 | Amide III | Rygula et al., 2013 [9]; Moskovits et al., 2021 [10] |
| 1294 | δ(CH_2_,CH_3_) | Rygula et al., 2013 [9] |
| 1314 | δ(CH_2_,CH_3_) | Rygula et al., 2013 [9] |
| 1370 | δCH_3_ | Rygula et al., 2013 [9] |
| 1423 | δCH_2_ | Zhu et al., 2011 [11] |
| 1450 | δ(CH_2_,CH_3_) | Rygula et al., 2013 [9]; Moskovits et al., 2021 [10] |
| 1464 | δ(CH_2_,CH_3_) | Rygula et al., 2013 [9], Moskovits et al., 2021 [10] |
| 1491 | His | Beier et al., 2007 [12] |
| 1600 | Phe | Rygula et al., 2013 [9] |
| 1650 | Amide I | Rygula et al., 2013 [9] |
| 1675 | Amide I | Rygula et al., 2013 [9] |

**Table S2**. Performances of the classifier in training set for both the machine learning analyses, i.e., AD patients vs. ONC (non-AD patients), and AD patients *vs.* HyC patients. We reported each score as mean value (standard deviation) over the iterations of the 5-fold cross-validation.

| **Dataset** | **AUROC** | **Accuracy** | **Sensitivity** | **Specificity** |
| --- | --- | --- | --- | --- |
| AD vs. ONC | 0.93 (0.03) | 0.94 (0.02) | 0.98 (0.01) | 0.86 (0.04) |
| AD vs. HyC | 0.92 (0.04) | 0.95 (0.03) | 0.99 (0.01) | 0.85 (0.07) |

AD: Alzheimer’s disease, ONC: other neurodegenerative conditions, AUROC: area under the receiver operating characteristic curve, HyC: patients with normal pressure hydrocephalus.

**REFERENCES:**

1. Banchelli M, Amicucci C, Ruggiero E, D’Andrea C, Cottat M, Ciofini D, et al. Spot-on SERS Detection of Biomolecules with Laser-Patterned Dot Arrays of Assembled Silver Nanowires. ChemNanoMat. 2019; 5:1036-1043.

2. Barucci A, D’Andrea C, Farnesi E, Banchelli M, Amicucci C, De Angelis M, et al. Label-free SERS detection of proteins based on machine learning classification of chemo-structural determinants. Analyst. 2021; 146(2):674-682.

3. Krafft C, Schmitt M, Schie IW, Cialla-May D, Matthäus C, Bocklitz T, et al. Label-Free Molecular Imaging of Biological Cells and Tissues by Linear and Nonlinear Raman Spectroscopic Approaches. Angew. Chemie - Int. Ed. 2017; 56(16):4392-4430.

4. D’Andrea C, Foti A, Cottat M, Banchelli M, Capitini C, Barreca F, et al. Nanoscale Discrimination between Toxic and Nontoxic Protein Misfolded Oligomers with Tip-Enhanced Raman Spectroscopy. Small. 2018; 14(36):e1800890.

5. Moores B, Drolle E, Attwood SJ, Simons J, Leonenko Z. Effect of Surfaces on Amyloid Fibril Formation. Sokolov I, editor. PLoS One. 2011;6:e25954.

6. Blackley HKL, Patel N, Davies MC, Roberts CJ, Tendler SJB, Wilkinson MJ, et al. Morphological development of β(1-40) amyloid fibrils. Exp Neurol. 1999; 158(2):437-43.

7. Podstawka E, Ozaki Y, Proniewicz LM. Part I: Surface-enhanced Raman spectroscopy investigation of amino acids and their homodipeptides adsorbed on colloidal silver. Appl Spectrosc. 2004; 58(5):570-80.

8. Roy A, Chandra K, Dolui S, Maiti NC. Envisaging the Structural Elevation in the Early Event of Oligomerization of Disordered Amyloid β Peptide. ACS Omega. 2017; 2(8):4316-4327.

9. Rygula A, Majzner K, Marzec KM, Kaczor A, Pilarczyk M, Baranska M. Raman spectroscopy of proteins: A review. J. Raman Spectrosc. 2013; 44:1061-1076.

10. Moskovits M, Piorek BD. A brief history of surface-enhanced Raman spectroscopy and the localized surface plasmon Dedicated to the memory of Richard Van Duyne (1945–2019). J. Raman Spectrosc. 2021; 52:279.

11. Zhu G, Zhu X, Fan Q, Wan X. Raman spectra of amino acids and their aqueous solutions. Spectrochim Acta - Part A Mol Biomol Spectrosc. 2011; 78(3):1187-95.

12. Beier HT, Cowan CB, Chou IH, Pallikal J, Henry JE, Benford ME, et al. Application of surface-enhanced raman spectroscopy for detection of beta amyloid using nanoshells. Plasmonics. 2007; 2:55–64.
